# Response to the letter to the editor: Thyroid surgery volume – A statement issued by the Brazilian Head and Neck Surgery Society (SBCCP)

**DOI:** 10.20945/2359-4292-2024-0066

**Published:** 2024-06-03

**Authors:** Laura Sterian Ward, Rafael Selbach Scheffel, Ana Oliveira Hoff, Carolina Ferraz, Fernanda Vaisman

**Affiliations:** 1 Universidade Estadual de Campinas Laboratório de Genética Molecular do Câncer Faculdade de Ciências Médicas Campinas SP Brasil Laboratório de Genética Molecular do Câncer, Faculdade de Ciências Médicas, Universidade Estadual de Campinas (Unicamp), Campinas, SP, Brasil; 2 Universidade Federal do Rio Grande do Sul Hospital de Clínicas de Porto Alegre Faculdade de Medicina Porto Alegre RS Brasil Unidade de Tireoide, ospital de Clínicas de Porto Alegre, Faculdade de Medicina Universidade Federal do Rio Grande do Sul, Porto Alegre, RS, Brasil; 3 Universidade Federal do Rio Grande do Sul Instituto de Ciências Básicas da Saúde Departamento de Farmacologia Porto Alegre RS Brasil Departamento de Farmacologia, Instituto de Ciências Básicas da Saúde, Universidade Federal do Rio Grande do Sul, Porto Alegre, RS, Brasil; 4 Universidade de São Paulo Instituto do Câncer do Estado de São Paulo Unidade de Oncologia Endócrina São Paulo SP Brasil Unidade de Oncologia Endócrina, Instituto do Câncer do Estado de São Paulo (Icesp), Universidade de São Paulo (USP), São Paulo, SP, Brasil; 5 Irmandade da Santa Casa de Misericórdia de São Paulo Divisão de Endocrinologia Departamento de Medicina São Paulo SP Brasil Divisão de Endocrinologia, Departamento de Medicina, Irmandade da Santa Casa de Misericórdia de São Paulo, São Paulo, SP, Brasil; Faculdade de Ciências Médicas da Santa Casa, São Paulo, SP, Brasil; 6 Instituto Nacional de Câncer Serviço de Oncologia Endócrina Rio de Janeiro RJ Brasil Serviço de Oncologia Endócrina, Instituto Nacional de Câncer (INCA), Rio de Janeiro, RJ, Brasil; 7 Universidade Federal do Rio de Janeiro Serviço de Endocrinologia Faculdade de Medicina Rio de Janeiro RJ Brasil Faculdade de Medicina, Serviço de Endocrinologia, Universidade Federal do Rio de Janeiro, Rio de Janeiro, RJ, Brasil

## DOES BRAZIL HAVE ENOUGH HIGH-VOLUME HEAD AND NECK SURGEONS?

**TO THE EDITOR:**

We read with great interest the letter by Matos and cols. (1) regarding our position statement published in 2022 (2). As rightly observed, the sentence pointed out in the letter reflects our opinion in the position statement, which is why the sentence was not supported by a reference. Our opinion, in turn, was based on arguments presented in the section where the sentence was inserted, which was supported by references (*e.g.*, a consensus by the American Thyroid Association [ATA]). The ATA consensus, based on two strong pieces of evidence, states that "Surgeon experience likely influences the risks of thyroidectomy, with higher volume surgeons having lower complication rates (232,233)" (3). The importance of high surgical volume in the outcomes of patients with thyroid disease is also endorsed in several publications (4-6) and incorporated into the recommendations of various guidelines and statements issued by important groups (7). Considering that Brazil is a vast country, high-volume head and neck surgeons (*i.e.*, 792 active members of the Brazilian Society of Head and Neck Surgery, according to data presented in the letter,) are mainly based in large cities and referral centers. For example, from 22,780 thyroid procedures performed within the private health care system, more than 16,000 (>70%) were carried out in only five Brazilian states: São Paulo, Rio de Janeiro, Minas Gerais, Pernambuco, and Santa Catarina (8). Thus, it is essential to consider that many of these procedures might have been performed either by less experienced head and neck surgeons, by surgeons who could not keep up with the minimum number since they left residency, or by general surgeons. Furthermore, a recent study analyzing public institutions in Brazil showed that 556 institutions performed 15,331 thyroidectomies in 2019. The institutions were then stratified according to the surgical volume into three categories: low volume (100 thyroidectomies/year), intermediate volume (10-100 thyroidectomies/year), and high volume (>100 thyroidectomies/year). Based on this categorization, 258 (46.4%) institutions were classified as low volume, 269 (48.4%) as intermediate, and 29 (5.2%) as high volume. In the same year, 848 (5.5%) thyroidectomies were performed in low-volume, 9,404 (61.4%) in intermediate-volume, and 5,079 (33.1%) in high-volume institutions. The proportion of thyroidectomies performed in high-volume institutions among all states in 2019 displayed a median of 28.5% (interquartile range 0.0-43.5%) (9). Still, an important point raised in the letter by Matos and cols. (1) is the need for further studies to better understand real-life surgical expertise throughout the country.
